# Integrating HIV advanced disease management into a routine program setting: cohort from Mumbai, India

**DOI:** 10.1186/s12913-025-12572-5

**Published:** 2025-04-24

**Authors:** Shrikala Acharya, Ramesh Reddy Allam, Vijay Kumar Karanjkar, Dhirubhai Rathod, Prashant Deshpande, Amol Palkar, Shashikant Todmal, Sagar Koli, Sachin Dhande, Nalini Chava, Vijay V Yeldandi, Amit Harshana, Reshu Agarwal, Sunita Upadhyaya, Melissa Nyendak

**Affiliations:** 1 Mumbai Districts AIDS Control Society, Acworth Complex, R.A. Kidwai Marg, Wadala West, Mumbai, Maharashtra India; 2 U.S. Centers for Disease Control and Prevention, Division of Global HIV and Tuberculosis (DGHT), Panchsheel Marg, Shantipath, Chanakyapuri, New Delhi, Delhi 110021 India; 3 International Training and Education HIV (I-TECH), 808, 8th Floor, Ansal Bhawan, 16, Kasturba Gandhi Marg, Connaught Place, New Delhi, India; 4 Society for Health Allied Research and Education (SHARE INDIA), Ghanpur Village, Medchal Malkajgiri District, Hyderabad, Telangana State India

**Keywords:** Advanced HIV disease, Advanced disease management, HIV survival, Mortality

## Abstract

**Background:**

The advanced disease management (ADM) package, which aims to reduce morbidity and mortality in people with Advanced HIV disease (AHD, WHO stage III/IV and/or CD4 count < 200 cells/mm^3^ or age < 5 years), is not fully implemented in India. We assessed the feasibility of implementing the full WHO ADM package as part of routine HIV care under the programmatic setting in antiretroviral therapy centers of Mumbai.

**Methods:**

We implemented the ADM package (screening, treatment, and prophylaxis for major opportunistic infections, rapid ART initiation, and ART adherence support) in 17 ART centers from October 2020 to December 2021. Treatment naïve and experienced persons with AHD, including children, were enrolled. We assessed the feasibility through coverage of ADM package components and reported the proportion of rapid ART initiation (≤ 7 days), cotrimoxazole prophylaxis, TB preventive treatment (TPT) for those eligible [(excluded active TB disease (*n* = 280) and those completed TPT prior to enrolment (*n* = 1,186)], TB-LAM screening (excluded current TB disease), and cryptococcal antigen (CrAg) assay (excluded children < 10 years of age). We used a point of care test for TB (LAM) and cryptococcus (CrAg) screening. We followed the prospective cohort for one year (through 31 July 2022) to document outcomes for survival and lost to follow- up (LTFU).

**Results:**

We identified 4,334 PLHIV with AHD and provided the full ADM package to 64% (2,779/4,334); 297 did not receive ADM (146 died, 151 LTFU), and 1,258 received routine standard of care (587 had TB, 366 were at decentralized sites, and 305 LAM/CrAg kits were not available) with existing ART center staff. Nearly 78% (385/494) of treatment naïve were rapidly initiated on ART. Nearly 82% (1,129/1,383) and 99% (2,751/2,779) received TPT and cotrimoxazole prophylaxis, respectively. Of the eligible, 99% (2,508/2,524) and 98% (2,715/2,758) were screened for TB and cryptococcal infection, respectively. At the end of 12 months, 88% (2,458/2,779) were alive, 8% (210/2,779) died, and 4% (111/2,779) were LTFU. Mean survival time was significantly (*p* < 0.001) higher among treatment experienced people; 11.6 months (95% CI: 11.5,11.7) compared to treatment naïve people 10.8 months (95% CI: 10.5,11.0).

**Conclusion:**

With careful anticipatory planning, stakeholder engagement, and training, implementing the full ADM package is feasible in a routine program setting with existing human resources. Additional intensive case management may be necessary for the reduction of mortality among treatment naïve PLHIV.

## Contributions to the literature

- Our findings suggest that, with careful planning and involving stakeholders from health providers and communities, it is feasible to implement continuity-of-care innovations such as the HIV ADM package at all levels of health facilities to improve the survival and outcomes of PLHIV.
- Efficient and person-centric referral mechanisms and additional intensive care are required for antiretroviral treatment naïve clients with AHD.
- Training, mentoring, and monitoring of health care staff with the development of person-centric advanced HIV disease patient flow systems are crucial for ADM implementation.

## Background

Expanded HIV testing and rapid access to antiretroviral therapy (ART) is a key pillar to reach the 2030 HIV elimination goals. Of the 39 million people living with HIV (PLHIV), an estimated 29.8 million are on life saving ART (2022), leading to a significant impact on reduction in mortality globally [1]. Despite ongoing improvements in ART access, advanced HIV disease (WHO stage III or IV and /or CD4 count < 200 cells/mm^3^ or age < 5 years) remains a significant cause of death among PLHIV [2, 3], in low- and middle-income countries. To decrease morbidity and mortality and expand evidence-based practice, WHO recommends an Advanced Disease Management (ADM) package for children [4] and adults [5]. The WHO ADM package components include screening and diagnosis of opportunistic infections (for active TB with Nucleic Acid Amplification Test and the lateral flow urine lipoarabinomannan (LAM) assay, systematic screening for serum or plasma cryptococcal antigen (CrAg) assay), cotrimoxazole prophylaxis, TB preventive treatment fluconazole primary prophylaxis, rapid ART initiation and intensive antiretroviral treatment adherence support.

India’s National AIDS Control Program has seen a decline in annual new HIV infections and AIDS related deaths by 86% and 78% (2021), with nearly 1.6 million PLHIV on ART [6] through expanded person-centric service delivery strategies, which have improved access and closed gaps in treatment continuity. Despite these advances, 30% of PLHIV newly presenting to care for enrollment in ART centers have advanced HIV disease [7] with CD4 counts < 200 cells/mm^3^. This necessitates a public health approach for managing people who present late with AHD [8, 9].

In 2020, with the recommendation of the National Technical Resource Group, we assessed the feasibility of implementing the full ADM package in the 17 government ART centers in the routine program setting in Mumbai. In 2021, India’s National AIDS Control Program reviewed the WHO guidance and drafted guidelines on Advanced Disease Management [10] for implementation at ART centers. Despite the broader adoption of the WHO guidance, there are barriers to implementation. Currently, the National AIDS Control Program implements a partial ADM package [4] that includes, symptom screening for TB followed by TPT for those negative screen, Nucleic Acid Amplification Test for TB diagnosis, cotrimoxazole prophylaxis] across government ART centers due to limited availability of TB-LAM and CrAg screening tests and fluconazole prophylaxis. Through an implementation science approach, we describe the ADM adoption and report barriers, moderators, and outcomes. The full results of TB LAM (included PLHIV > 18 years of age) and CrAg implementation referred to in this paper are reported elsewhere [11, 12].

### Methods

From October 2020–December 2021, we implemented the WHO recommended full ADM package into routine HIV services in 17 public antiretroviral therapy centers in Mumbai. Six centers were in tertiary, eight in secondary, and three in primary health care facilities. Care provided at these ART centers is free of charge to the PLHIV. As described in our prior study, “ART centers consist of a team of different cadres of health care providers including a trained physician, nurse, pharmacist, counsellor, data manager, lab technician, and a community representative. In the programmatic setting, CD4 count is enumerated for all PLHIV at baseline and every six months while on ART, and viral load is done annually. ART centres refer PLHIV to higher health facilities for diagnostic or clinical management, if required” [11, 12].

To describe the process of translating the ADM package into routine service delivery at ART centers, we adopted Per Nilsen’s [13] implementation science theoretical approach that stratifies different theories based on the setting, process, and outcomes. We used the Ottawa Model of Research [14] approach to describe the process of integration of the ADM package into routine care at ART centers in Mumbai and have described the implementation through the six-step approach of the Ottawa Model. This approach is most suitable for implementing continuity-of-care innovations that involve multiple sectors, settings, agencies, and provider groups [15] that impact the adoption of changes at the organizational and systems level [16].

### Application of the six-step approach of the Ottawa model for implementation of the ADM package

#### Step 1. Stakeholder engagement

Mumbai is one of India’s three mega Metropolitan cities, with a well-organized separate administrative body (the Mumbai Corporation). Apart from the routine budgetary allocation from the National AIDS Control Organization, the Mumbai Corporation was a critical stakeholder in resource mobilization and facilitation of policy. We included clinicians, microbiologists, program managers, community members, clinicians, administrators of hospitals and ART center staff as key stakeholders. We organized a series of consultative meetings with stakeholders to seek inputs on operational aspects, standard operating procedures (SOP) on care and treatment, patient flow, referral mechanisms, reporting, and monitoring tools.

#### Step 2. Components of ADM package of services

We developed SOPs and trained ART center staff on the ADM package as per the SOPs. The WHO ADM package [17] includes (1) rapid ART initiation (within 7 days), (2) prophylaxis and pre-emptive treatment including cotrimoxazole prophylaxis, TPT, provided TB disease is excluded by initial four symptom screening (4 S negative) and among 4 S positive TB was ruled out by nucleic acid amplification test and or TB-LAM and or chest radiography, and fluconazole pre-emptive therapy of 800 mg/day for 2 weeks, then 400 mg/day for 8 weeks and continued maintenance with fluconazole 200 mg/day for serum CrAg antigen using in vitro CrAg Lateral Flow Assay (Immy, Norman, OK, USA) positive people without meningitis, (3) expanded diagnostic options with TB-LAM assay using the in vitro Alere1 Determine TB LAM Antigen lateral flow assay (Abbott Laboratories, Lake Bluff, USA) to add to TB disease screening with Nucleic Acid Amplification Test and prompt initiation of anti-TB treatment and the systematic screening with serum cryptococcal antigen assay (excluded children < 10 years) and (4) intensive treatment adherence support during routine pill-pick-up visits by the counsellor to achieve 95% adherence and telephonic reminders for pill-pickup.

PLHIV who were not provided with an ADM package were provided with a routine standard of care that included other components of ADM such as screening and diagnosis of active TB with Nucleic Acid Amplification Test, cotrimoxazole prophylaxis, TB Preventive therapy, rapid ART initiation and intensive treatment adherence support along with CD4 and viral load monitoring which are a part of the routine program.

#### Step 3. Potential barriers and facilitators for implementing the ADM package

Although there were ADM guidelines at the national level, we did not have SOPs at the site level. Other site level barriers included the lack of guidance on rapid ART initiation, limited availability of drugs required for preventive therapy [cotrimoxazole, fluconazole, and isoniazid], and absence of urine LAM and serum CrAg assays in India’s National AIDS Control program. Additionally, and mirroring the impact of COVID-19 globally, during April–July 2021 there was a surge in the number of persons with COVID-19 across India; Mumbai was disproportionately hit with disruption in transportation services and other critical services including health. We received approval to procure a limited number of TB-LAM kits for use under research purpose as urine TB-LAM is not approved for clinical use in India. Despite these challenges, we identified several facilitators. Through strong political will and support from the Mumbai Districts AIDS Control Society (MDACS), the Society committed to the availability and forecasting of ADM package including drugs, consumables, and laboratory kits, along with the staff time at the ART centers. Additionally, we relied on an existing strong network of interdepartmental referrals and a robust lab-clinic interface with the ART center, in addition to existing monitoring, site mentoring and review mechanisms as facilitators for uptake of ADM implementation in Mumbai.

#### Step 4. Intervention

**Fig. 1:**
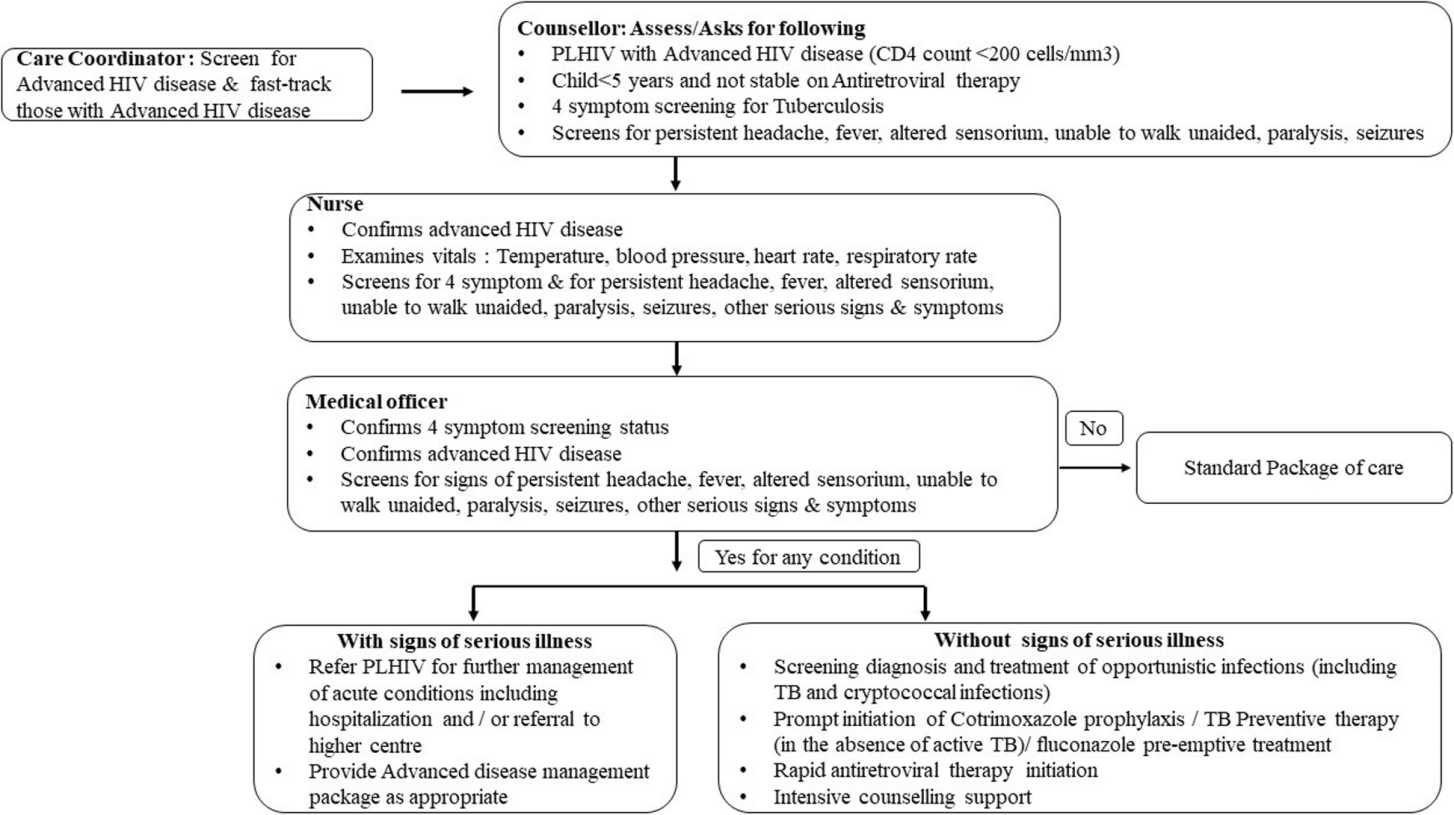
Operational flow of advanced disease management services at ART centers, Mumbai

We enrolled all adolescents and adults who had current CD4 counts < 200 cells/mm3 or WHO clinical stage III / IV events, regardless of whether they were ART-naive or ART-experienced. We also enrolled all children < 5 years of age not established on ART and enrolled children who have been receiving ART for > 1 year and were clinically unstable. We trained the ART center staff to ensure uniform implementation and monitoring of PLHIV in the ADM package of services. We developed job aids and standard operating procedures (Fig. 1) for use in the ART center upon identification of a person with AHD and ensured ART and laboratory staff adhere to operational and technical aspects of ADM management. We provided hands-on training on the laboratory workflow, emphasizing the pre-analytical procedures for performing LAM and CrAg testing to the nurse and lab technician of ART centers. ART center staff assessed all PLHIV during their routine ART center visit for eligibility and ensured the provision of the ADM package and recorded details in the ADM register.

#### Step 5. Assess the adoption of the ADM package

Mumbai District AIDS Control Society provided on-site supportive supervision, clinical mentoring, and monitoring support to the ART center staff.

The ART facilities were assessed monthly for the availability of drugs, kits, and other consumables. During the monthly mentoring and monitoring on-site visits to the ART centers, the staff observed the process of enrollment of eligible PLHIV into the ADM package, reviewed registers for accuracy, and provided specific recommendations and course corrections on referral and management of PLHIV. Mumbai Districts AIDS Control Society routinely reviewed ART center performance every quarter based on key implementation measures e.g., number of PLHIV presenting to ART center with AHD, number of newly initiated PLHIV presenting with AHD, the number enrolled into ADM package, the number offered with specific components.

#### Step 6. Outcomes

We reported feasibility, program, and client outcomes.

### Feasibility outcomes

Coverage of each component of the ADM package was assessed to determine the feasibility of implementing the ADM package. We calculated the proportion of PLHIV provided with ADM package of care among those presenting with AHD, the proportion of treatment naïve PLHIV rapidly initiated on ART, the proportion of eligible PLHIV initiated on TPT of those screened negative for TB [excluded persons with current TB disease and those having completed TPT prior to ADM enrolment], the proportion of eligible PLHIV initiated on cotrimoxazole prophylaxis, the proportion of PLHIV offered urine TB-LAM (excluded current TB disease), the proportion of PLHIV offered serum cryptococcal antigen (CrAg) assay (excluded children of < 10 years of age) and proportion offered ART adherence counselling. We reported the methods and results of the operational feasibility of implementing TB-LAM and CrAg assay elsewhere [11, 12].

### Program outcomes

We defined a turnaround time of two hours for receipt of urine TB-LAM and serum CrAg results. We calculated the median turnaround time for both urine TB-LAM and serum CrAg. Clients who received the ADM package were followed for one year (through 31 July 2022) to document outcomes (alive on ART, died, lost to follow-up). We calculated the mortality rate using the number of deaths as the numerator and the duration of treatment (in person-years) as the denominator (including those LTFU and died). Data was censored on 31 July 2022. We used the Kaplan-Meier product limit estimation method to assess the cumulative probability of survival. The log-rank test was used to examine the statistical difference in survival by type of PLHIV (treatment experienced and treatment naïve).

### Client outcomes

We calculated the proportion of PLHIV screened positive for urine TB-LAM and serum CrAg. The Cox proportional hazard regression model was used to calculate the hazard ratios for death as a primary outcome at any time during the follow-up period to determine predictors of mortality. Variables with p value of 0.2 or below and having biological relevance to the outcome were included in the final model.

### Operational definitions

We defined ‘alive on ART’ as PLHIV receiving ADM package of services, picked up ART within 28 days of pill-pick date, and alive on care as of 31 July 2022; ‘died’ as PLHIV on ART who received ADM package but died due to any cause at the end of one-year follow-up and lost to follow-up (LTFU) as PLHIV who missed pill pick up for > 28 days at the end of one year of follow-up.

### Data collection, data management, and analysis

Routine program data was collected from the patient records (treatment card, TB register, and ART register) maintained at the ART center. Variables such as age, sex, ART status (treatment experienced and ART naïve), duration of ART, CD4 count, serum and cerebrospinal fluid CrAg results, urine LAM results, follow-up outcomes (alive on ART, died, lost to follow-up), TPT initiation status, anti-TB treatment initiation status, cotrimoxazole prophylaxis were collated. All data were entered in Microsoft Excel (Version MSO (16.0.10383.20027). We calculated the mean, standard deviation, median, and interquartile range (IQR) for continuous variables, and proportions for categorical variables. We analyzed data using IBM SPSS Statistics for Windows (Version 23.0).

## Results

**Fig. 2:**
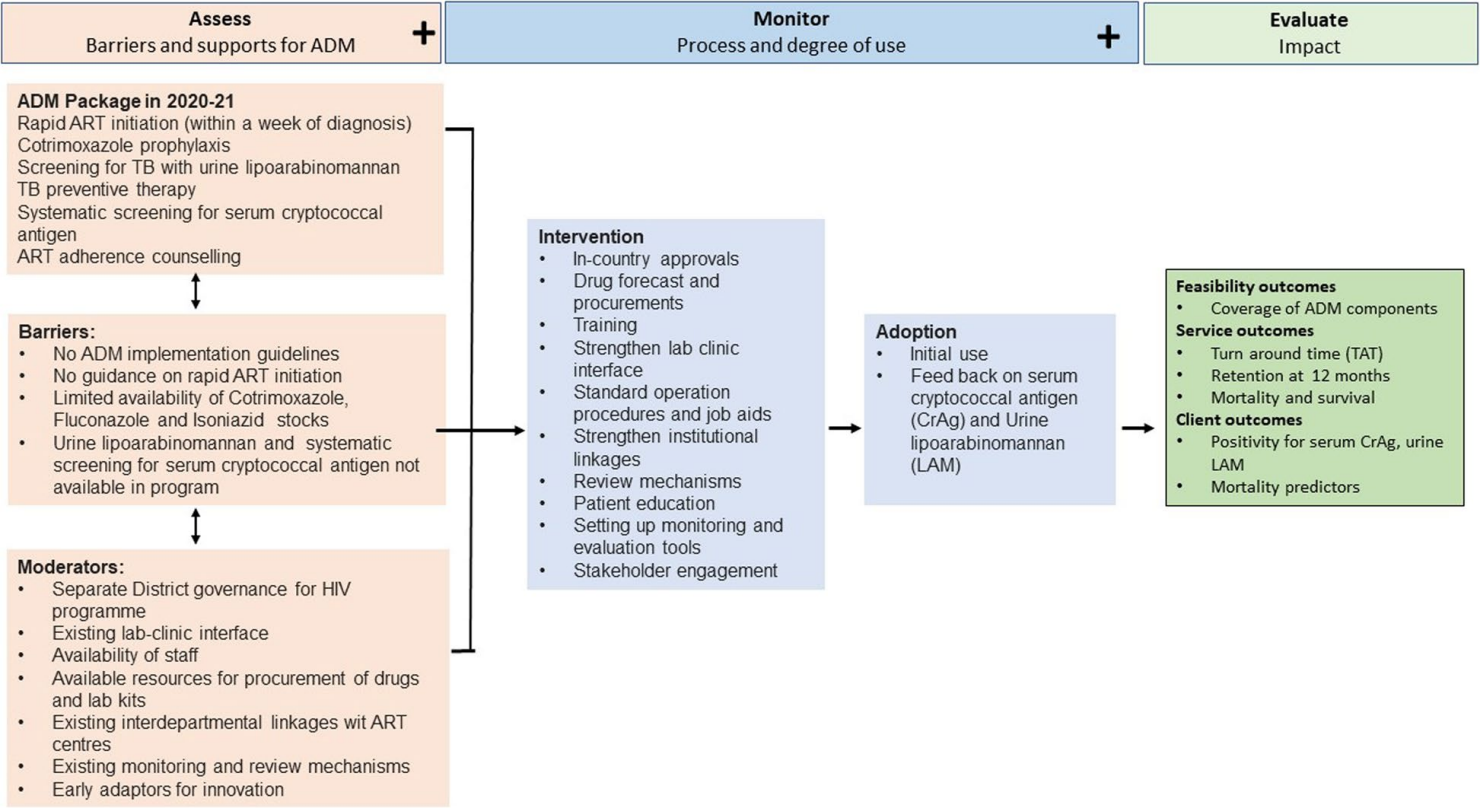
Ottawa model for implementation of advanced disease management package of services, Mumbai

We categorized the results based on the Ottawa model into assess, monitor, and evaluate (Fig. 2).

### Assess

All the cadre of staff from 17 ART centers were trained on the ADM package of services. We provided 2,779 AHD persons with ADM package with existing staff at the ART center. Of those who received the ADM package, 68.1% (1,892) were males, the median age was 43 years [IQR: 35,50], and the majority (35.8%) were between 40 and 49 years of age. The median CD4 count was 137 cells/mm^3^ [IQR: 80, 218], and 34.5% (959) presented with CD4 count ≤ 100 cells/mm^3^. The majority, 79.2% (2,212/2,779), were treatment experienced, and 82.8% (2,302/2,779) did not have symptoms of TB on four symptom screening (Table 1).

**Table 1:** Demographic, immunological, and clinical characteristics of AHD clients provided with advanced disease management package of services, Mumbai (*N* = 2,779)

Characteristics		*n* (%)
Age in years	<20	107 (3.9)
	20–39	863 (31.1)
	40–59	1632 (58.7)
	≥ 60	177 (6.4)
Sex	Male	1892 (68.1)
	Female	887 (31.9)
Type of population	Key Population	80 (2.9)
	General Population	2699 (97.1)
WHO clinical stage at enrolment	I	2045 (73.5)
	II	349 (12.6)
	III	180 (6.5)
	IV	205 (7.4)
CD4 Count (cells/mm^3^) at enrolment	≤ 50	214 (7.7)
	51–100	471 (16.9)
	101–200	1914 (68.9)
	> 200	180 (6.5)
Viral load (copies/ml) at enrolment	< 1000	1328 (47.8)
	≥ 1000	536 (19.3)
	Not Available	915 (32.9)
4 symptom (4 S) screening for TB	4 S Positive	202 (7.3)
	4 S Negative	2302 (82.8)
	Presented with TB	275 (9.9)
Duration on ART	Newly initiated on ART (ART naïve)	567 (20.4)
	≤ 1 year of prior ART	306 (11.0)
	> 1 to < 2 Years of prior ART	309 (11.1)
	> 2 Years of prior ART	1597 (57.5)

Key Population (KP) includes female sex workers, people with injecting drug use, and men who have sex with men. KP data was abstracted from routine program records

### Monitor

We forecasted the supply of drugs such as cotrimoxazole, fluconazole, and isoniazid to avoid stockouts during the implementation and follow-up period. The Mumbai District’s AIDS Control Society team conducted a monthly review with the ART center staff to review processes, including the lab clinical interface to ensure linkage and referral services for case management. The monthly review provided a real time opportunity to review progress while providing solutions for challenges with refresher training and through on-site mentoring to fast-track ADM implementation. During the initial quarter of implementation, there were challenges, such as identifying the PLHIV with AHD during the visit to ARTC and timely providing the ADM package by the ART center staff.

### Evaluate

#### Feasibility outcomes

**Fig. 3:**
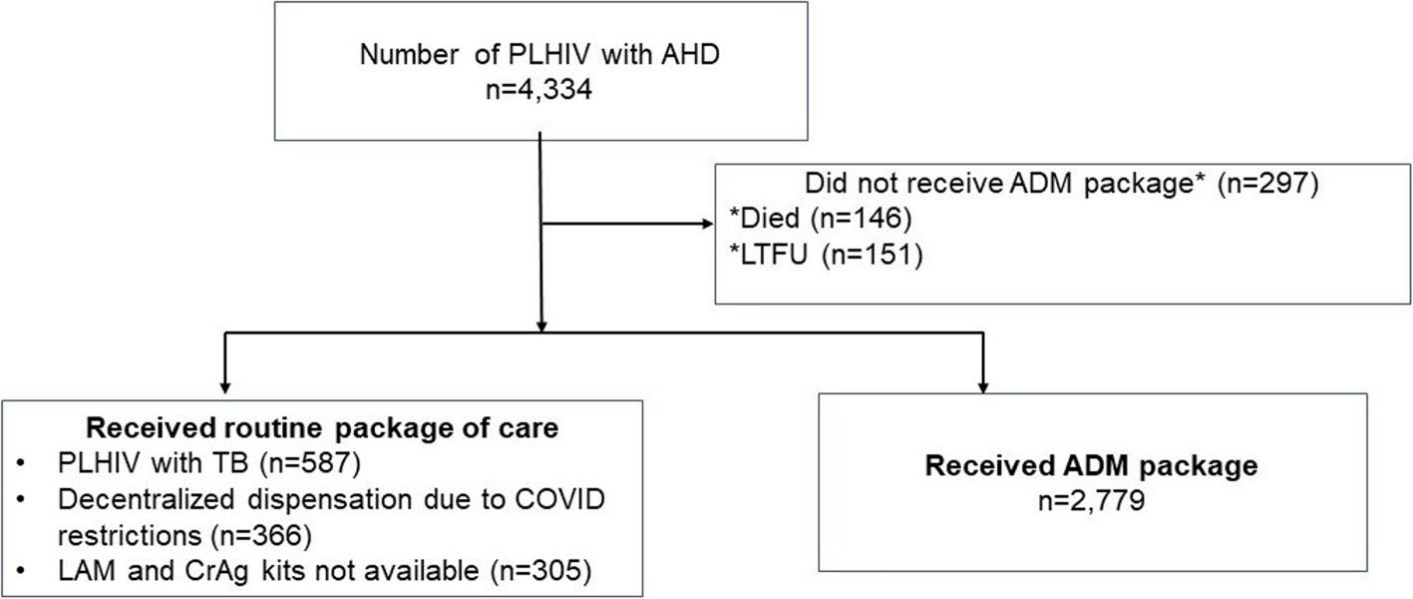
PLHIV provided with ADM package and routine care, Mumbai

We identified 4,334 PLHIV who qualified for ADM services. During October 2020 to December 2021, we were able to provide the full package of ADM services to the majority who qualified (64%; 2,779/4,334) using existing staff. Of the 1,555 PLHIV who could not be provided with the full ADM package, 297 did not receive the ADM package (146 died, 151 LTFU) and 1,258 received routine standard of care (587 had TB, 366 were at decentralized sites, and 305 LAM/ CrAg kits were not available) with existing staff at ART centers (Fig. 3).

Of the treatment naïve PLHIV, 77.9% (385/494) were rapidly initiated (< 7 days) on ART. After excluding persons with TB disease (*n* = 280) and those having completed TPT prior to ADM enrolment (*n* = 1,186), 81.6% (1,129/1,383) received TPT and 99.0% (2,751/2,779) received cotrimoxazole prophylaxis. Of the eligible, 99.4% (2,508/2,524) and 98.4% (2,715/2,758) were screened for TB and cryptococcal infection by TB-LAM and CrAg Point of care test, respectively (Table 2).

**Table 2:** Feasibility of implementing components of advanced disease management package of services

Characteristics	*N* (%)
**Rapid Antiretroviral therapy (ART) initiation**	
Eligible PLHIV (ART naïve)	494
Rapid initiation of ART	385 (77.9%)
**Tuberculosis preventive treatment (TPT)**	
Eligible for TPT	1,383
Initiated TPT	1,129 (81.6%)
**Cotrimoxazole prophylaxis**	
Eligible for Cotrimoxazole prophylaxis	2,779
Initiated Cotrimoxazole prophylaxis	2,751 (99.0%)
**Tuberculosis screening and disease diagnosis**	
PLHIV with AHD	2,779
PLHIV presented with concurrent TB disease	274
Eligible for TB screening	2,505
Total screened for TB (4 symptom screening)	2490 (99.4%)
Total screened positive on 4 S screening	234 (9.3%)
Sputum Xpert tested among 4sysmptom positive	137
Xpert positive among 4 symptoms positive	28 (20.4%)
Urine TB LAM positive among 4 symptoms positive	45 (19.2%)
Both Xpert and LAM positive among 4 symptoms positive	24 (10.3%)
Total screened Positive using (LAM/ Xpert / both) among 4 symptoms negative clients	
Xpert tested among 4 symptoms negative	13
Xpert positive among 4 symptoms negative	4 (30.8%)
Urine TB LAM positive among 4 symptoms negative	126 (5.6%)
Total Urine TB LAM positive among those screened for 4 symptoms	171 (6.8%)
**Cryptococcal infection screening by Cryptococcal antigen (CrAg)**	
Eligible for CrAg screening	2,758
Total screened for CrAg	2,715 (98.4%)
Total CrAg positive	25 (0.9%)
Proportion referred for Cerebrospinal fluid CrAg	92.0% (23/25)

#### Program outcomes

All PLHIV screened for TB and or cryptococcal infection by urine TB-LAM and serum CrAg received test results within a turnaround time of 2 h. At the end of 12 months, among those provided with the ADM package, 88.4% (2,458/2,779) were alive, 7.6% (210/2,779) died, and 4.0% (111/2,779) were LTFU (Table 3). Of the 1,555 who could not be provided with the full ADM package, 297 did not receive an ADM package nor routine standard of care (146 died, 151 LTFU) and 1,258 received routine standard of care. Of the 1,258 who received routine standard of care, 80% (1001/1,258) were alive, 14% (178/1,258) died, and 6% (79/1,258) were LFTU.

**Table 3:** Twelve-month outcomes of clients with advanced HIV disease provided with ADM package of services, Mumbai (*N* = 2,779)

Characteristics	*N*	%
Alive on ART	2,458	88.4
Died	210	7.6
Lost to follow-up	111	4.0

**Fig. 4:**
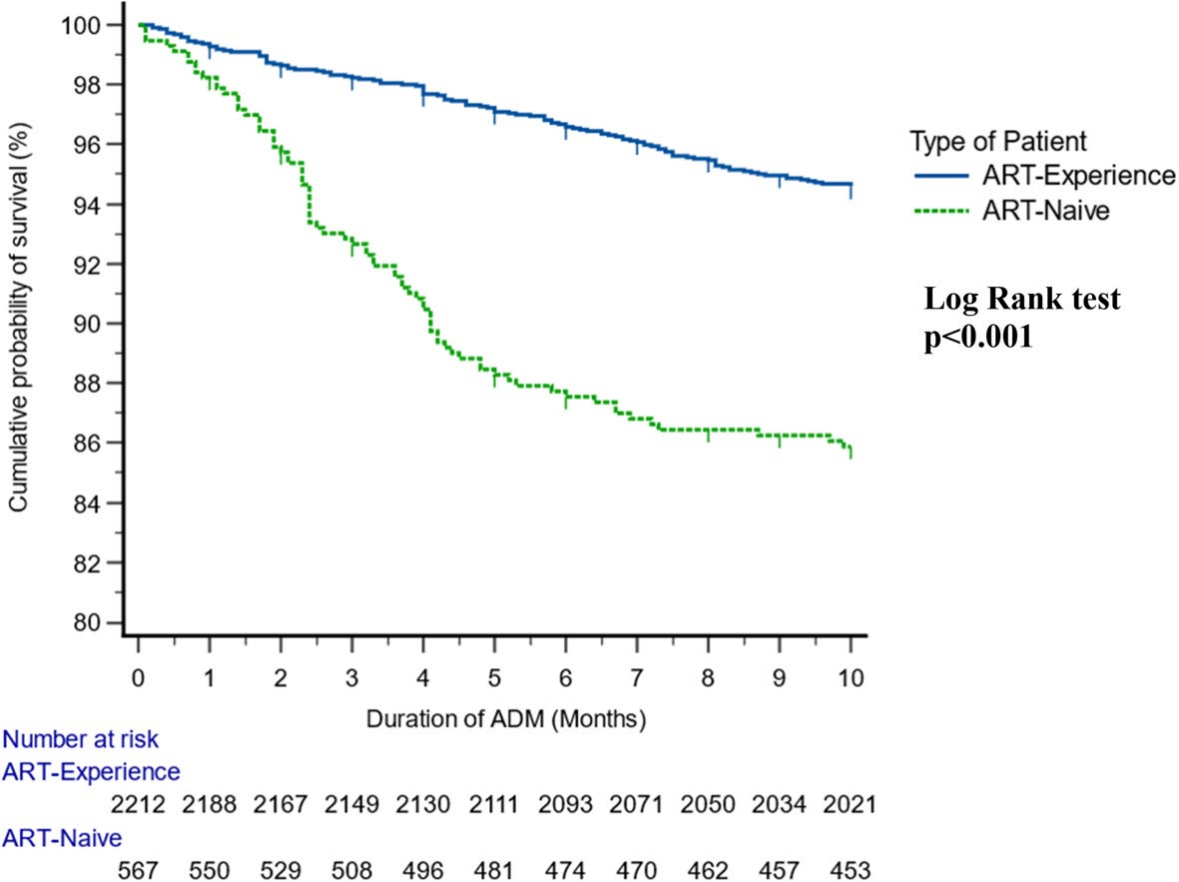
Cumulative survival probability of PLHIV with Advanced HIV Disease by type of PLHIV, Mumbai Kaplan-Meier curves displaying the estimated survival probability for 2 different groups of patients (ART-Experience & ART-Naïve). Each vertical step in the curve indicates one or more events (i.e., deaths), and right censored patients are indicated by a vertical mark in the curve at the censoring time. A visual inspection suggests that survival seems to be more favorable for ART-Experienced patients than ART-Naïve patients. The log-rank test indicates a significant difference between the survival curves

The overall mean survival of the cohort was 11.4 months (95% CI: 11.30,11.47) at 12 months of follow-up. Mean survival time was significantly (*p* < 0.001) higher among treatment experienced 11.6 months (95% CI: 11.53,11.69) compared to treatment naïve 10.8 (95% CI: 10.51,11.03) at 12 months (Fig. 4).

The overall mortality rate of the cohort was 8.1 per 100-person years (210/2,596). The mortality rate at 90 and 180 days after treatment initiation was 3 per 100-person years and 7 per 100-person years, respectively. Of the 210 total deaths in the ADM group, 161 (76.6%) had occurred during the first 6 months after the ADM package was started. Of the 161 PLHIV who died within six months on ADM package of care, about 60% were between 40 and 59 years of age, the majority were female (66%), 51% presented with a CD4 count less than 100 cells/mm3, 26% had TB, and 45% were treatment naïve.

#### Client outcomes

##### Predictors of mortality

PLHIV aged 40–59 years (95%CI: 1.21, 13.97; *p* < 0.02) and ≥ 60 years (95%CI: 2.02, 24.88; *p* < 0.002), not initiated on TPT (95%CI: 1.53, 2.97; *p* < 0.001), serum CrAg positive (95%CI: 1.16, 7.0; *p* < 0.022), viral load ≥ 1000 (95%CI: 32.91,243.76; *p* < 0.001), without viral load after ADM enrolment (95%CI: 34.90, 261.98; *p* < 0.001), and treatment naïve (95%CI:1.08,2.05; *p* < 0.014) had higher risk of death compared to those with age < 20 years, initiated on TPT, serum CrAg negative, viral load < 1000 copies/ ml, and treatment experienced respectively (Table 4).

**Table 4:**
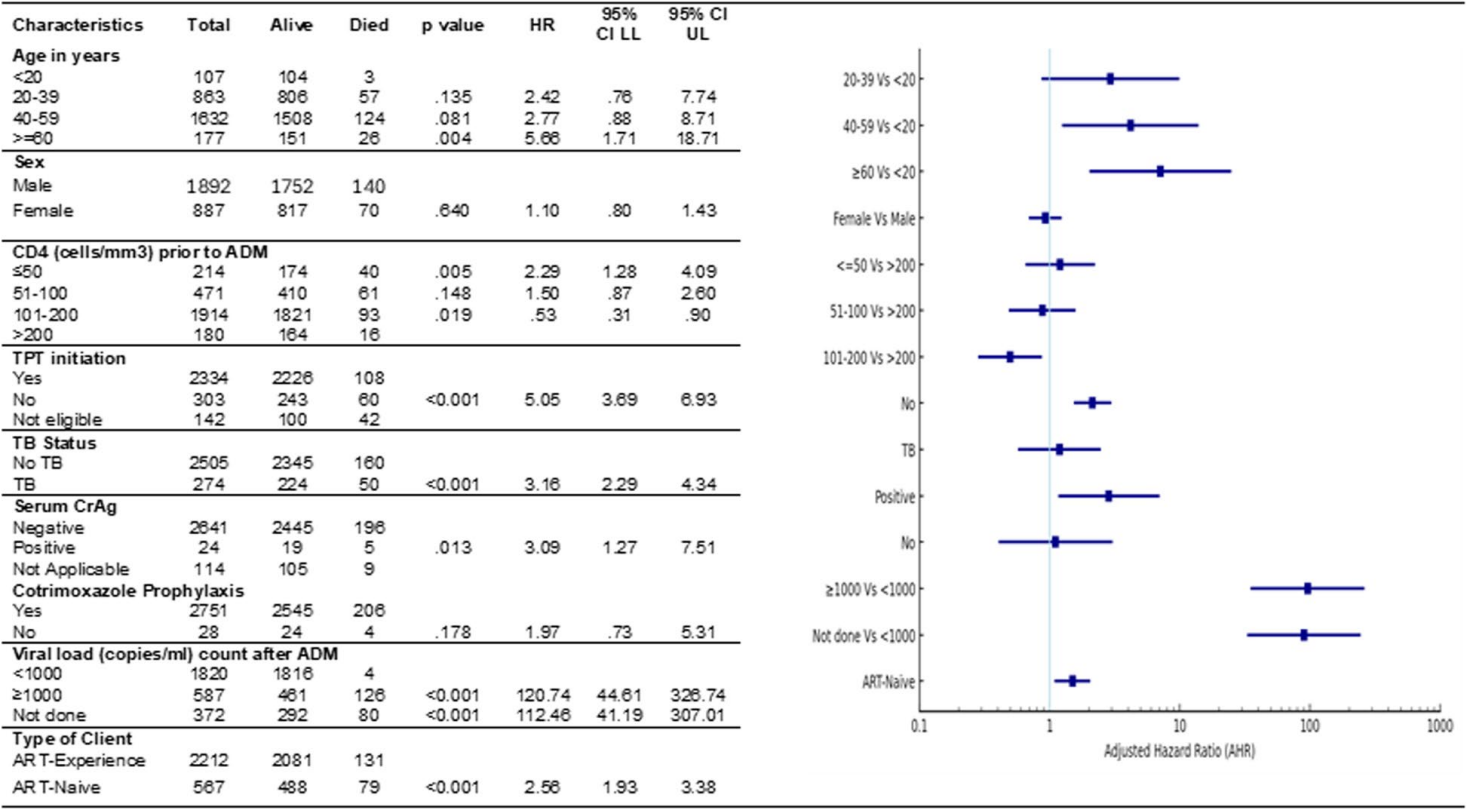
Predictors of mortality among clients with advanced HIV disease provided with advanced disease management package of care, Mumbai (*N* = 2,779)

##### TB-LAM and CrAg positivity

As reported previously [11, 12], a total of 2,508 PLHIV were offered urine TB-LAM. The overall proportion of PLHIV with urine TB-LAM positive result regardless of TB symptoms was 6.8% (171/2,508), and 91.8% (157/171) were initiated on anti TB treatment (Table 2). Among PLHIV with CD4 ≤ 100 cells/mm^3^, the positivity was 43.0% and 7.7% in symptomatic and asymptomatic patients, respectively. Among PLHIV with a CD4 > 100 cells/ mm^3^, the positivity was 26.7% and 2.7% in symptomatic and asymptomatic patients respectively. Of the eligible 2,758, 98.4% (2,715) were tested for serum CrAg. Of these, 25 (0.9%) had a CrAg positive result. Among CrAg positive patients, only one had symptoms (Table 2). Lumbar puncture was obtained in 20/25 patients within 4 days (range: 1–4 days) of positive serum CrAg result, and one person was confirmed to have meningitis. All serum CrAg-positive patients who had a negative cerebrospinal fluid CrAg were offered fluconazole pre-emptive therapy of 800 mg/day for 2 weeks, then 400 mg/day for 8 weeks, and continued maintenance with fluconazole 200 mg/day.

## Discussion

We report on a large prospective cohort from India receiving the full WHO recommended ADM package of care and prospectively followed for twelve months to assess the feasibility and intervention outcomes in the government program setting. Implementing the ADM package in Mumbai entailed engaging several stakeholders, community members, healthcare providers, and patients in a real-world setting [16]. Most of the cohort provided with the ADM package of services in this analysis were treatment experienced, and about 60% were aged between 40 and 59 years. The increased representation of PLHIV in this age band is also observed at the national level. Per the national HIV estimates for 2022, about 36% of the total PLHIV were estimated to be in the age group of 50 years or older, which is significantly different from 2000 where only around 17% of the total PLHIV were aged 50 years or older [6].

During the planning stage, and because country specific guidance for ADM implementation was not available, we developed job aids, regular on-site clinical mentoring, hands-on laboratory training, and monitoring to inform the implementation process for the ADM package by the clinical and laboratory staff. During the initial quarter of implementation, there were challenges, such as the identification of PLHIV with AHD during the visit to the ART center and the timely providing the ADM package. Regular review, monitoring, and mentoring support during the initial implementation was vital to ensure a uniform ADM package of services. As described in prior studies, task sharing, training, and mentorship improved the uptake of laboratory and clinical ADM services among nurses and counsellors [17, 18]. We observed that the coverage of all ADM components did not depend on the patient volume (high or low) of PLHIV or the location (tertiary or secondary) of the ART centers. In fact, with anticipatory planning, implementation was possible without additional human resources.

Since ART centers are a part of the secondary or tertiary institutions, establishing linkage and referral systems for investigations, higher level clinical management, and strengthening lab-clinic interface are essential prerequisites for successful ADM implementation in a routine program setting. We established strong referral pathways that enabled serum CrAg positive PLHIV to access lumbar puncture services at a higher center in an inpatient setting. We trained and mentored staff on a job aid and algorithm to ensure appropriate investigation and higher-level clinical management when indicated in other studies [19]. In our intervention, the government stakeholders undertook anticipatory forecasting of drugs and lab commodities, human resource management, and capacity building in the planning stage leading to the high screening coverage of point of care test (TB-LAM (99%) and CrAg (96%). Similar to the experience from South Africa, in our study, the point of care test minimized the testing burden on central laboratories and provided results within a turnaround time of 2 h [20].

WHO recommends urine TB-LAM [21] among AHD patients for early TB diagnosis and prevention of mortality. Our study found an additional yield when TB-LAM was coupled with the standard program recommended diagnostics and documented the utility and feasibility in the program setting. However, clinical use is limited in India and other high HIV-TB burden countries [22, 23]. CrAg lateral flow assay is approved for patient management in India, but the National AIDS Control program may consider access at all ART centers for reflexive screening of all PLHIV with CD4 count < 100 cells / mm^3^ [24]. The ART center staff in Mumbai adapted to routinely screen PLHIV for AHD and continue providing the requisite ADM service package. Implementation of evidence-based practices from prior studies has indicated that adaptations are often made by systems, organizations, and/or service providers in the implementation process [25].

Rapid ART initiation is a critical component of ADM: however, in our study, we could achieve 78% coverage (84 had TB, 8 had serum positive CrAg and 17 died even before the clinical assessment for ART initiation). Similar barriers to rapid ART initiation are described in prior studies [26]. Similarly, TPT coverage was 83% and this lower rate could be attributed to the national program recommendation of delayed TPT initiation after three months of ART [27].

The 12-month mortality among PLHIV on ADM package was 8.1 per 100 person years; the majority (72%, 161/224) occurred within 6 months of ART initiation, and the mean survival was higher among the treatment experienced group compared to the treatment naive. Similar to our findings, a review of the meta-analysis and cohort studies from middle- and high-income countries in 2021, indicated a higher risk of mortality during the first six months [28]. Even after initiating ART, people with advanced immunosuppression have a 50% higher rate of mortality as compared to people with a CD4 count > 200 cells/ mm^3^ at the time of ART initiation [29]. WHO recommends additional close monitoring or intensive case management procedures such as. screening and diagnostic testing for major opportunistic infections and, where possible, access to radiology and laboratory services to be provided to assist with diagnosis and monitoring of PLHIV with severe AHD for a reduction in mortality [30].

Similar findings were observed in studies done in Ethiopia and Uganda wherein the higher odds of mortality rate were higher among PLHIV with CD4 < 200 mm^3^, viral load ≥ 1000 copies/ml, and advanced disease patients (stage III and IV), not initiated or not eligible for TPT [31, 32]. In the program setting, we describe a reduction in mortality with the ADM package as similar to that of the REMSTART [33] and Reality trial [34]. We also note that the National AIDS Control Program NACP introduced dolutegravir based regimens as the preferred ARV, which could have additionally contributed to the reduction in mortality.

### Limitation

Our prospective cohort study followed people during the COVID-19 pandemic and LTFU and mortality could have been additionally impacted. Within the program setting, we may not have measured all opportunistic infections among patients presenting with AHD.

## Conclusion

With careful anticipatory planning, stakeholder engagement, and training, implementing the full ADM package is feasible in a routine program setting with existing human resources. Availability of point of care tests allowed for additional diagnostic and clinical management options for patients with AHD. Efficient up-referral, including the development of person-centric advanced HIV disease patient flow systems, training and mentoring of health care staff, and monitoring are also crucial for ADM implementation. Additional intensive case management may be necessary for the reduction of mortality among treatment naïve PLHIV with AHD.

## Data Availability

There are ethical and legal restrictions on sharing the de-identified data set. As these data are related to PLHIV containing sensitive information, there are restrictions as per the National law (HIV prevention and control act, 2017). The ethics committee secretary Dr. Shivkar can be reached by email: mdacsec@gmail.com for data request.

## Abbreviations

ADM: Advanced Disease Management
AHD: Advanced HIV Disease
ART: Antiretroviral Therapy
CrAg: Cryptococcal Antigen
Lf-LAM: Lateral flow lipoarabinomannan
LTFU: Lost to follow-up
PLHIV: People Living with HIV
SOP: standard operating procedures
TPT: TB Preventive Therapy
